# Supplementary material for: The Relation between Hair-Cortisol Concentration and Various Welfare Assessments of Dutch Dairy Farms
Source: Animals (Basel). 2021 Mar 15;11(3):821. doi: 10.3390/ani11030821 (PMC7998858; doi:10.3390/ani11030821)
Supplement: Supplementary file 1 [file animals-11-00821-s001.pdf]

animals

MDPI

Supplementary Material

The Relation Between Hair-Cortisol Concentration and Various Welfare Assessments of Dutch Dairy Farms

Frank J. C. M. van Eerdenburg [1,*], Tessa Hof [2], Benthe Doeve [2], Lars Ravesloot [2], Elly C. Zeinstra [2], Rebecca E. Nordquist [2] and Franz Josef van der Staay [2]

1 Department of Farm animal Health, Faculty of Veterinary Medicine, Utrecht University, Yalelaan 7, 3584 CL Utrecht, The Netherlands
2 Behaviour and Welfare Group, Faculty of Veterinary Medicine, Utrecht University, Yalelaan 7, 3584 CL Utrecht, The Netherlands; t.hof518@gmail.com (T.H.); benthedoeve@gmail.com (B.D.); lars.ravesloot@wur.nl (L.R.); e.c.zeinstra@uu.nl (E.C.Z.); r.e.nordquist1@uu.nl (R.E.N.); f.j.vanderstaay@uu.nl (F.J.v.d.S.)
* Correspondence: f.j.c.m.vaneerdenburg1@uu.nl

Citation: van Eerdenburg, F.J.C.M.; Hof, T.; Doeve, B.; Ravesloot, L.; Zeinstra, E.C.; Nordquist, R.E.; van der Staay, F.J. The Relation between Hair-Cortisol Concentration and Various Welfare Assessments of Dutch Dairy Farms. *Animals* **2021**, *11*, x. https://doi.org/10.3390/xxxxx

Academic Editor: Paul Koene

Received: 30 January 2021
Accepted: 10 March 2021
Published: date

Publisher's Note: MDPI stays neutral with regard to jurisdictional claims in published maps and institutional affiliations.

Copyright: © 2021 by the authors. Submitted for possible open access publication under the terms and conditions of the Creative Commons Attribution (CC BY) license (http://creativecommons.org/licenses/by/4.0/).

Tables with the elements measured in all the protocols used.

Table S1. Welzijnswijzer (WW) (=Welfare Indicator)

Number of animals
present at the farm
with BCS < 1.5 *
lying in alleys
with swollen hocks
< size of a fist
> size of a fist
with swollen carpal joints,…
< size of a fist
> size of a fist
with arthritis (swollen, painful joint)
with thickness on dorsal neck
with dirty hindquarters, size of the dirty area
25 × 25–50 × 50 cm
50 × 50cm–½ hindquarter
> ½ hindquarter
with skin infections with fungi
with scabies
with lesions
with clinical mastitis
with impaired teat condition (any type of injury or damage)
with claw score 3 (angle > 24º)
that are severely lame
with lameness score 1 (Sprecher score)
with lameness score 2 & 3
with lameness score 4 & 5
with avoidance test > 2m
with avoidance test < 2m, but not touched
with avoidance test touched
Number of places at the feed rack
Height of the feed rack
Width of a feeding place at the feed rack
Quality of the drinking water
Access to the drinking water
Bedding is soft (non-painful kneetest)
Quantity of bedding
Number of lying places
Length of free stall
Width of free stall
Number with open front
Number with closed front
Diagonal (distance of neck rail—curb)

* Wildman, E.E.; Jones, G.M.; Wagner, P.E.; Dirkzwager, A.; Boman, R.L.; Troutt Jr., H.F.; Lesch, T.N. A dairy cow body condition scoring system and Its relationship to selected production characteristics. *J Dairy Sci* **1982**, *65*, 495–501.

Table S2. Continuous Welfare Monitor (CWM)

Percentage cows with somatic cell count > 250,000
Economic result
Bulk milk cell count
Percentage dead cows (per annum)
Non return < 56 days
Expected calving interval

Table S3. Koekompas (KK) (=Cow Compass) (welfare related parameters)

Activity / fearfulness
BCS < 2 and > 4 *
Locomotion score 3,4,5 (Sprecher system)
Hock score
Hygiene
Deviant cows, rumination, manure, rumen score
General impression, hair, skin lesions
High somatic cell count (number of animals)
Clinical mastitis
Claw disorders
Metabolic disorders
Retentio secundinarum
Vaginitis
Embryonic deaths
Forced culling
Other diseases
number of places at the feed rack
height of the feed rack
width of a feeding place
Softness of bedding
quantity of bedding
number of lying places
length of free stall
width of free stall
Head space
Cleanliness of the free stalls
Access to pasture
Quality of the path to pasture
Barn environment: Light, ventilation, mechanical brush.
Quality of the feed
Does the feed fit the group?
Quality and quantity of drinking water

* Wildman, E.E.; Jones, G.M.; Wagner, P.E.; Dirkzwager, A.; Boman, R.L.; Troutt Jr., H.F.; Lesch, T.N. A dairy cow body condition scoring system and Its relationship to selected production characteristics. *J Dairy Sci* **1982**, *65*, 495-501.

Table S4. Stall Standing Index (SSI)

Percentage of cows standing with 2 or more feet in the stall

Table S5. Cow comfort scoring system (CCSS)

**General**	**Concentrate Dispenser**
Number of cows standing idle	Number
Fear behavior	Type (period-/saving system)
Stretching when raising from cubicle	
Tail is hanging straight and relaxed	**Water**
Bellowing	Number of places
Cows lying in walkways	Type of waterer
Noise (environmental)	Cleanliness
	Temperature
**Light**	
Sufficient light in the barn	**Waiting room and milking parlor**
Period of light > 15 h	Behavior
Period of dark > 6 h	Time spent every milking
**Ventilation**	**Walkways and alleys**
It smells fresh (between the animals)	Width of the alley behind the feeding fence
Cobwebs	Width other walkways
Condense/mold	Sufficient passages
Barn temperature	
Dead spaces	**Miscellaneous**
Draft	Maternity pen
	Sick bay
**Cubicles/Free stalls**	Access to pasture / outside paddock
Cows are clean	Is there a mechanical brush?
Bedding is made of inorganic material	
Bedding is soft	**Animal health + feeding**
Bedding is clean and dry	Hair
Stall surface is under a slight angle	Lameness
Bedding is flat	Hocks
Neck rail	Claws
Lunge space	Mastitis
Stall dimensions	Abomasal displacement
Brisket board	Filling of the rumen
Number	Milking fever
	Acetonaemia (Ketosis)
**Floor**	BCS
Slipperiness	Fat %
Loose/unequal slats	Fertility
Rubber	Calving
Walking	
Cleanliness	
**Feeding fence**	
Headlocks (yes or no)	
Height	
Number of places	
Contamination of feed	

Table S6. New Welfare Monitor (WM)

Principle	Parameters Measured
Feed & water	% very lean cows
	Water supply
Housing	Free stall dimensions
	Softness of bedding (with knee test)
	Cleanliness of the cows
	Access to pasture (as in WQ)
	Cows lying outside free stall (as in WQ)
Health	Locomotion score (Sprecher system)
	Skin lesions (as in WQ)
	% mastitis (milk somatic cell count > 400,000)
	Other diseases (respiratory/metabolic/fertility) (as in WQ)
Behaviour	Avoidance distance at the feeding fence (as in WQ)
	Possibilities for expression of normal behaviour (as in WQ)

Table S7. Welfare Quality (WQ)

Number of lactating cows	
Type of housing (loose vs tied)	
**Feeding**	**Behaviour**
% very lean cows	Frequency of butts per cow per hour
Number/length/flow of water troughs sufficient?	Frequency of other aggressive events
At least 2 water bowls available for each cow	% cows that can be touched
Are the drinkers clean?	% cows that can be approached by 50 cm but not touched
**Housing**	% cows that can be approached between 50 cm and 1 m
Duration of lying down movements (in sec)	% cows that can't be approached
% lying down movements with collisions	Tendency to be active
% lying cows which lie partly outside lying area	Tendency to be relaxed
% cows with dirty lower legs	Tendency to be fearful
% cows with dirty udder	Tendency to be agitated
% cows with dirty flank and upper legs	Tendency to be calm
Number of days with access to OLA per year	Tendency to be content
Number of hours with access to OLA per day	Tendency to be indifferent
Number of days on pasture per year	Tendency to be frustrated
Number of hours on pasture per day	Tendency to be friendly
**Health**	Tendency to be bored
% not lame cows	Tendency to be playful
% moderately lame cows	Tendency to be positively occupied
% severely lame cows	Tendency to be lively
% cows with no integument alterations	Tendency to be inquisitive
Number of hairless patches per cow	Tendency to be irritable
Number of lesions + swellings per cow	Tendency to be uneasy
Frequency of coughing per cow per 15 min	Tendency to be sociable
% cows with nasal discharge	Tendency to be apathetic
% cows with ocular discharge	Tendency to be happy
% cows with increased respiratory rate	Tendency to be distressed
% cows with diarrhoea	
% cows with vulvar discharge	
% mastitis (milk somatic cell count > 400,000)	
% mortality during the last 12 months	

% dystocia
% downer cows
% dehorned cows
Method used for dehorning
Use of anaesthetics for dehorning
Use of analgesics for dehorning
% tail-docked cows
Method used for tail-docking
Use of anaesthetics for tail docking
Use of analgesics for tail docking

Table S8. Modified Welfare Quality (WQ Mod)

Number of lactating cows	
Type of housing (loose vs tied)	
**Feeding**	**Behaviour**
% very lean cows	Frequency of butts per cow per hour
Number/length/flow of water troughs sufficient?	Frequency of other aggressive events
At least 2 water bowls available for each cow	% cows that can be touched
Are the drinkers clean?	% cows that can be approached by 50 cm but not touched
**Housing**	% cows that can be approached between 50 cm and 1 m
Duration of lying down movements (in sec)	% cows that can't be approached
% lying down movements with collisions	
% lying cows which lie partly outside lying area	
% cows with dirty lower legs	
% cows with dirty udder	
% cows with dirty flank and upper legs	
Number of days with access to OLA per year	
Number of hours with access to OLA per day	
Number of days on pasture per year	
Number of hours on pasture per day	
**Health**	
% not lame cows	
% moderately lame cows	
% severely lame cows	
% cows with no integument alterations	
Number of hairless patches per cow	
Number of lesions + swellings per cow	
Frequency of coughing per cow per 15 min	
% cows with nasal discharge	
% cows with ocular discharge	
% cows with increased respiratory rate	
% cows with diarrhoea	
% cows with vulvar discharge	
% mastitis (milk somatic cell count > 400,000)	
% mortality during the last 12 months	
% dystocia	
% downer cows	
% dehorned cows	
Method used for dehorning	
Use of anaesthetics for dehorning	

Use of analgesics for dehorning
% tail-docked cows
Method used for tail-docking
Use of anaesthetics for tail docking
Use of analgesics for tail docking
